# Cancer Genetics and Therapeutic Opportunities in Urologic Practice

**DOI:** 10.3390/cancers12030710

**Published:** 2020-03-18

**Authors:** Jacob J. Adashek, Alex Leonard, Jason Roszik, Arjun K. Menta, Giannicola Genovese, Vivek Subbiah, Pavlos Msaouel

**Affiliations:** 1 Department of Internal Medicine, University of South Florida, H. Lee Moffitt Cancer Center & Research Institute, Tampa, FL 33606, USA; jadashek@westernu.edu; 2 Morsani College of Medicine, University of South Florida, Tampa, FL 33612, USA; alexleonard@health.usf.edu; 3 Departments of Genomic Medicine and Melanoma Medical Oncology, The University of Texas MD Anderson Cancer Center, Houston, TX 77030, USA; jroszik@mdanderson.org; 4 The University of Texas at Austin, Austin, TX 78712, USA; arjunkmenta@gmail.com; 5 Department of Genitourinary Medical Oncology, Division of Cancer Medicine, The University of Texas MD Anderson Cancer Center, Houston, TX 77030, USA; ggenovese@mdanderson.org; 6 Department of Investigational Cancer Therapeutics, Division of Cancer Medicine, The University of Texas MD Anderson Cancer Center, Houston, TX 77030, USA

**Keywords:** cancer genetics, urologic oncology, urology, next-generation sequencing

## Abstract

This article aims to summarize the current literature on genetic alterations related to tumors of the genitourinary tract. Novel associations have recently been reported between specific DNA alterations and genitourinary malignancies. The most common cause of chromosome 3p loss in clear cell renal cell carcinoma is a chromothripsis event, which concurrently generates a chromosome 5q gain. Specific patterns of clear cell renal cell carcinoma metastatic evolution have been uncovered. The first therapy targeting a specific molecular alteration has now been approved for urothelial carcinoma. Germline mutations in DNA damage repair genes and the transcription factor HOXB13 are associated with prostate cancer and may be targeted therapeutically. The genetic associations noted across different genitourinary cancers can inform potential screening approaches and guide novel targeted treatment strategies.

## 1. Introduction

**Figure 1:**
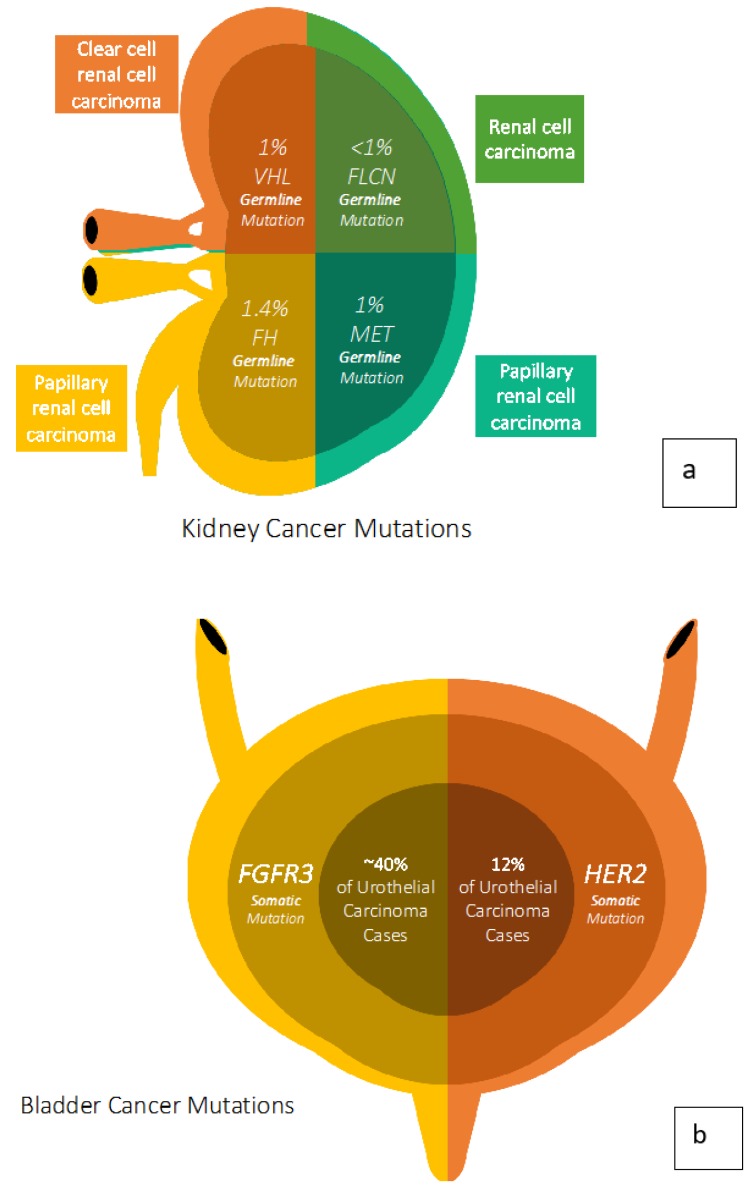

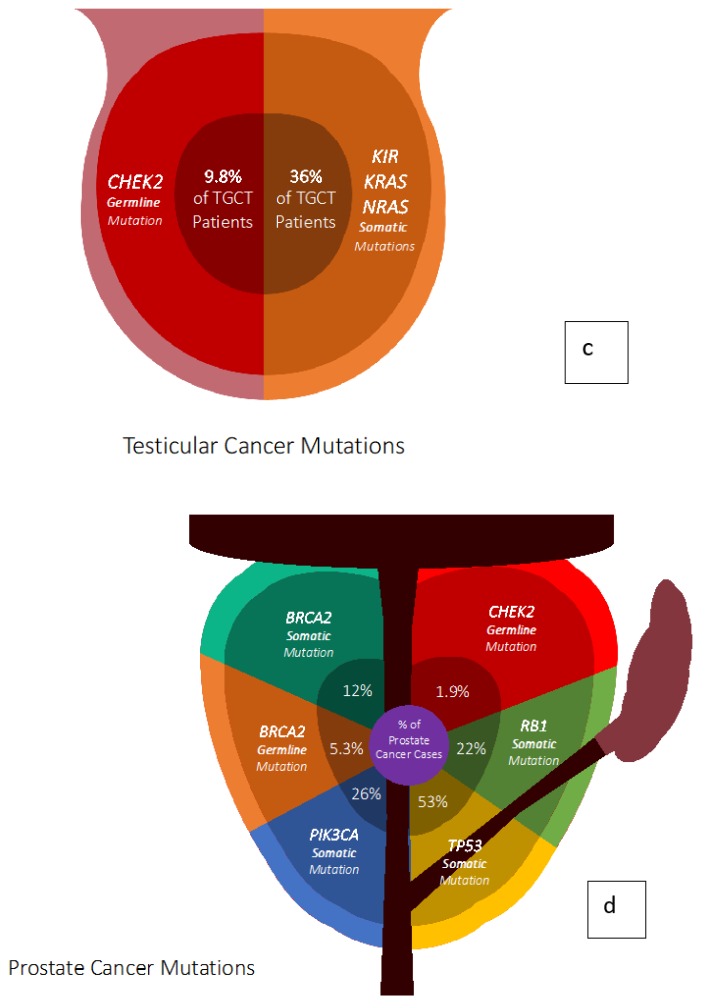
Germline and Somatic mutations found across tumor types. (**a**) Kidney Cancer Mutations, (**b**) Bladder Cancer Mutations, (**c**) Testicular Cancer Mutations, (**d**) Prostate Cancer Mutations.

Much of what is known about genitourinary (GU) cancers derives from the well-characterized genetic alterations in various hereditary syndromes (Figure 1). Using hereditary syndromes as assumptive models for sporadic, somatic disease processes allows investigators to make valuable biological and therapeutic inferences. Herein, we summarize recent developments in cancer genetics related to GU malignancies.

In renal cell carcinoma (RCC), von Hippel Lindau (VHL) disease, Birt–Hogg–Dubé (BHD) syndrome, and hereditary papillary renal carcinoma (HPRCC) have provided substantial insight into the pathogenesis of spontaneous RCC. Clear cell RCC (ccRCC), the most common form of RCC, is associated with alterations in the *VHL* gene causing a cascade of events, ultimately increasing the expression of vascular growth factors (VEGF). BHD is associated with activation of the *mTOR* genes in various types RCC, and HPRCC is known for its association to the *MET* gene [1,2,3]. All these pathways are well-described in RCC and can be therapeutically targeted [4].

Bladder cancers can be divided into low-grade and high-grade urothelial carcinomas, with each having distinct genetic aberrations. Mutations in *FGFR3* or *HRAS* are found in 65%–80% of low-grade cases and are less frequent in high-grade tumors, which are more likely to harbor mutations in *TP53* or *Rb* [5]. Understanding these key genomic alterations is paramount in recognizing the diversity of biology in bladder cancer. Other implicated pathways include *PIK3CA-mTOR* as well as *BAP1* [6]. The landscape of genomic alterations in bladder cancer and the intricate roles these mutations play in tumor proliferation can guide clinically effective treatment modalities. Recently, the first targeted therapy for urothelial carcinomas, erdafitinib, was approved by the FDA for the treatment of tumors harboring *FGFR2* and *FGFR3* alterations [7].

Germline mutations in the transcription factor *HOXB13* and DNA damage repair genes such as *BRCA1*, *BRCA2, CHEK2*, as well as the mismatch repair (MMR) genes *MSH6* and *PMS2,* have been shown to increase the risk of prostate cancer, the most common cancer among men [8,9,10,11,12,13]. For patients with *BRCA1*, *BRCA2,* and *ATM* alterations, there is now an FDA breakthrough designation for the use of olaparib, a poly ADP-ribose polymerase (PARP) inhibitor, in metastatic castration-resistant prostate cancer (mCRPC) [14]. Similarly, immunotherapy (IO) with pembrolizumab is now recommended by the National Comprehensive Cancer Network guidelines for MMR-deficient mCRPC [15].

In testicular germ cell tumors (TGCT), major genes associated with pathogenesis are *TP53* and its regulator *MDM2* in both seminomas and nonseminomas [16]. Although these are not specific to testicular cancer, their high oncogenicity has allowed further exploration into genomic biomarkers. In TGCT, there is growing evidence that *DNAAF1* mutations can also play a significant role in tumorigenesis [17]. Delineating molecular subtypes of testicular cancers can elucidate more genomic alterations and inform patient decision making.

## 2. Kidney Cancer Genetics 

A phase II study of pazopanib in 31 patients with molecularly confirmed or clinically consistent VHL disease demonstrated an objective response rate (ORR) of 42% in VHL-associated tumors (RCC, pancreatic lesions, and hemangioblastomas) pointing towards the clinical utility of pazopanib in this setting [18]. This is the first systemic therapy to show such encouraging efficacy in patients with VHL disease.

In the context of hereditary papillary RCC (HPRCC), the defining *MET* mutation has informed the design of various trials in sporadic papillary RCC with MET inhibitors. Treatment with MET inhibitors may lead to better outcomes in patients with MET-driven vs MET-independent papillary RCC [19]. Molecular insights into the role of *MET* in HPRCC informed the design of ongoing clinical trials such as SWOG1500 trial, which originally compared the VEGF inhibitor sunitinib to three different MET inhibitors (cabozantinib, crizotinib, and savolitinib) for the treatment of papillary RCC [20].

In Birt–Hogg–Dubé (BHD) syndrome, individuals are often afflicted with skin tumors, lung disease, and chromophobe RCC due to mutations in *FLCN* [21] leading to the downstream activation of mTOR, via the loss of negative inhibition by the BHD protein, similarly to how TSC1 and TSC2 complexes downregulate mTOR activity [21]. Patients with *FLCN* mutations and subsequent BHD, can provide valuable clinical insights on how chromophobe RCC will respond to the inhibition of the Akt-mTOR pathway [22]. In addition to modeling Akt-mTOR altered RCC, there is also growing evidence that hypoxia-inducible factor (HIF) is upregulated in *FLCN*-deficient RCC [23]. Increased levels of HIF lends itself to be targeted via HIF inhibitors, which are currently being evaluated in clinical trials. The loss of *FLCN* may warrant the dual blockade of Akt-mTOR and HIF pathways, which are both independent pathologic events in RCC.

In almost all (>90%) clear cell RCC (ccRCC) cases, the initial pathogenetic event is the loss of the 3p chromosome arm, which harbors the *VHL* gene [24]. The TRACERx Renal study recently reported that the most common mechanism of 3p loss in both sporadic and VHL-associated ccRCC is a chromothripsis event, which generates a concurrent gain of the 5q chromosome arm [25]. The same group found by analyzing 575 primary and 335 metastatic RCC samples that 87% of clonal variants in metastases are the same as in the primary tissue. Of the variants found in metastatic sites, only 5.4% were de novo mutations in driver genes such as *VHL*, *BAP1*, and *mTOR* [26]. Metastatic sites demonstrated different characteristics based on whether they harbored mutations in either *BAP1* or *PBRM1*. BAP1-driven tumors were characterized by increased tumor heterogeneity as well as high genomic instability and may thus be vulnerable to immunotherapeutic targets. On the other hand, PBRM1-mutated cases demonstrated more indolent clinical behavior and may benefit from cytoreductive nephrectomy [27]. Germline mutations in the *BAP1* gene have also been associated with BAP1-tumor predisposition syndrome which carries an increased risk of developing uveal melanoma, cutaneous melanoma, malignant mesothelioma, RCC, meningioma, and cholangiocarcinoma. Some groups recommend that, in patients who develop RCC at <46 years old, germline testing for *BAP1* mutations may identify earlier BAP1-tumor predisposition syndrome patients and offer better surveillance [28]. Another study found that among 181 families afflicted with BAP1-tumor predisposition syndrome, there were 140 unique germline variants in the *BAP1* gene [29]. This study found that 97.5% of missense variant carriers developed a *BAP1*-associated tumor, of which ~12% were RCC [29].

An interesting subset of kidney cancer is renal medullary carcinoma (RMC), which is a rare RCC subtype that predominantly affects young African Americans with sickle cell trait or other sickle hemoglobinopathies [30,31]. The *SMARCB1* (otherwise known as *INI1*, *BAF47*, or *SNF5*) tumor suppressor gene plays a key role in the pathogenesis of RMC, and all cases of RMC are defined by the loss of SMARCB1, as evidenced by immunohistochemistry [30]. RMC is a very aggressive form of RCC with poor overall survival following diagnosis, and shares some similarities to pediatric malignant rhabdoid tumors, which are also caused by *SMARCB1* inactivation [32]. A common mechanism of SMARCB1 loss is inactivating translocations [30], and a recently proposed mechanism of RMC pathogenesis postulates that regional ischemia induced by red blood cell sickling in the renal medulla of individuals with sickle cell trait or other sickle hemoglobinopathies can activate aberrant DNA damage repair mechanisms that can drive deletions and translocations in *SMARCB1*, which is located within a highly fragile region of chromosome 22 [33]. Of note, RMC is resistant to the standard VEGF-directed therapies used for ccRCC and other RCC subtypes. A study of mosaic mouse models with inactivated *SMARCB1* demonstrated that SMARCB1-negative tumors such as RMC are vulnerable to proteasome and autophagy blockade [34]. The sensitivity of RMC to proteasome inhibitors was further validated in a study of RMC cell lines [35]. An ongoing trial (NCT03587662) is thus now evaluating the efficacy of ixazomib, a potent proteasome inhibitor, in combination with gemcitabine and doxorubicin in patients with RMC and other SMARCB1-negative kidney malignancies.

Fumarate hydratase (FH) mutations are associated with aggressive papillary type 2 renal cell carcinoma termed FH-deficient renal cell carcinoma (FH-RCC) [36,37]. The majority of FH-RCC cases are associated with germline FH mutations as part of the hereditary leiomyomatosis and renal cell carcinoma (HLRCC) syndrome characterized by cutaneous and uterine leiomyomas and increased risk of FH-RCC [36,38]. FH is a tricarboxylic acid (TCA) cycle enzyme and thus FH-RCC tumors are characterized by impairment of the TCA cycle and of oxidative phosphorylation with a resultant metabolic shift to aerobic glycolysis which can be therapeutically targeted by the combination of erlotinib (inhibitor of cell membrane glucose transporters) with bevacizumab (inhibitor of glucose delivery via tumor neovasculature) [36].

## 3. Bladder Cancer Genetics

In individuals diagnosed with bladder cancer, the odds ratio (OR) that another family member had a history of bladder cancer was 2.34 (95% CI, 0.95–5.77) [39]. Whether this association is due to sharing a genetic driver or disease, or because certain shared family lifestyles may predispose an individual to develop bladder cancer is still unknown. Furthermore, in families afflicted by Lynch syndrome, the risk of developing urothelial carcinoma (UC), particularly of the upper tract, is significant [40]. The risk of developing UC before age 70 in men was 7.5% (95% CI, 3.1–11.9%) and in women 1.0% (95% CI, 0–2.4%) [39]. These findings support the notion that there is a familial genetic component to developing UC.

Sex differences are notable in bladder cancer as men are at higher risk of developing bladder cancer, whereas women are diagnosed at a later stage and have higher stage-adjusted mortality [41]. Furthermore, female patients with non-invasive bladder cancer are approximately two-fold more likely than men (74% vs. 42%) to harbor KDM6A mutations [42]. This may be in part because KDM6A is located on the X chromosome. The male paralogue of *KDM6A* is the *UTY* gene located on Yq11 and is mutated in approximately 9% of male non-invasive bladder cancers [42].

Other pathogenic drivers have been identified in bladder cancer and multiple trials to target these aberrations are ongoing. Up to 40% of primary UC have *FGFR3* mutations [43]. Patients with metastatic UC harboring *FGFR3* mutations, have shown to have minimal to modest responses to IO. However, patients can have meaningful benefit when particular FGFR3 alterations are targeted with an inhibitor. Specifically, the FGFR inhibitors rogaratinib and erdafitinib have shown ORRs between 40.4–59% in patients who progressed on IO [44]. Erdafitinib recently became the first targeted therapy approved for metastatic UC harboring *FGFR3* or *FGFR2* mutations [7].

The FGFR3 surface receptor activates a cascade of events that lead to activation of Ras and the PIK3CA pathway. Mutations in FGFR3 and Ras can occur in UC but are likely mutually exclusive events in carcinogenesis, with Ras mutations noted in about 11% of UC cases [45,46]. Mutations in PIK3CA have been found in 24% of UC cases and can co-exist with FGFR3 mutations in 15% of UC cases, making combined targeted therapy an attractive approach [46,47].

Another appealing target in bladder cancer has been human epidermal growth factor receptor 2 (HER2), which is found in about 12% of UC cases [48]. HER2-directed therapies have shown significant survival advantages in multiple cancer types, including breast, gastric, and esophageal. Interestingly, a phase III HER2-directed trial using lapatinib in UC found no benefit in HER2-positive UC patients compared with placebo following progression on first-line chemotherapy [49]. Other trials have tested trastuzumab in combination with chemotherapeutics, but so far none has demonstrated a substantial positive impact on survival outcomes [50]. There continue to be ongoing trials investigating the role of HER2-directed treatments for patients with HER2 mutations, but as of yet the utility of this mutation as a biomarker of benefit to directed therapy remains unknown.

## 4. Prostate Cancer Genetics

The heritability of prostate cancer has been analyzed for close to 30 years, and some models attribute roughly 9% of prostate cancers by age 85 to be genetic in nature [51]. An analysis of over 100,000 Nordic twins found that the heritability of prostate cancer development was 58% (95% CI, 52–63%) [52]. This confirmed a previously published study on World War II veteran twins that reported genetic heritability of prostate cancer accounting for 57% of cases [53]. 

Mutations in DNA repair genes such as *BRCA1*, *BRCA2*, *ATM*, *CHEK2*, and *PALB2* are of importance in prostate cancer. In a study of 82 patients, 11.8% had germline mutations in DNA repair genes namely *BRCA2* (5.3%), *ATM* (1.6%), *CHEK2* (1.9%), *BRCA1* (0.9%), and *PALB2* (0.4%) [54]. There is growing evidence that a substantial number of patients may benefit from therapies targeting these alterations. Other studies have found that individuals with germline *BRCA2* mutations are three-fold to 8.6-fold more likely to develop high risk prostate cancer [55,56]. Looking into *BRCA1*, a study of 813 cases of prostate cancer found that having *BRCA1* germline mutations resulted in a 3.75-fold relative risk for developing prostate cancer and in a cumulative risk of 8.6% of developing prostate cancer by 65 years old [57]. Additionally, comparing prostate cancer in germline *BRCA*-mutated (gBRCAm) cases to sporadic prostate cancer, a study of 2019 patients found that gBRCAm is associated with Gleason ≥8, T3/T4 stage, lymph node involvement, and metastatic disease at diagnosis [58]. This study also reported significantly worse cancer-specific survival in these patients compared to the noncarrier cohort, which was also validated in a different study [58,59], confirming the important role of genetics in prostate cancer development and prognosis.

Mutations in *BRCA1/2* and *ATM* can be targeted with PARP inhibitors. There are currently four FDA-approved PARP inhibitors (olaparib, rucaparib, niraparib, talazoparib) for other gBRCAm cancers including ovarian and breast [60,61,62,63,64,65]. These drugs have shown significant survival advantages in gBRCAm patients and previous approvals in other tumor histologies have served as the scientific basis for the currently ongoing PARPi trials in prostate cancer [66,67].

Analysis of familial risk models has revealed the importance of *HOXB13* G84E mutations in the development of early-onset prostate cancer [13]. A study in over 6000 patients, found that *HOXB13* gene mutations were significantly more likely in individuals with prostate cancer compared to those without (OR 20.1; 95% CI, 3.5–803.3) [13]. A follow-up meta-analysis looking at 25 case-control studies with a total of over 145,000 patients confirmed the increased risk for prostate cancer in *HOXB13* G84E mutant carriers (OR 3.248; 95% CI, 2.313–4.560; *p* < 0.001) [68]. It remains to be demonstrated whether regular screening for *HOXB13* G84E mutations in men with strong family history of prostate cancer can result in improved outcomes.

The *CHEK2* gene, which has been well-established in breast cancer pathogenesis, also has ties to prostate cancer. A study including over 86,000 patients found that heterozygotes with *CHEK2* mutations, were significantly more likely to develop prostate cancer than noncarriers (OR 1.60; 95% CI, 1.00–2.56) [69]. In familial cases, *CHEK2* mutations are associated with an increased risk of prostate cancer (OR 3.39; 95% CI, 1.78–6.47) [70]. Additionally, a 45,000-patient case-control study on specific subset populations found that African men and European men with *CHEK2* mutations has increased risk for developing prostate cancer (OR 3.03; 95% CI, 1.53–6.03; *p* = 0.0006 and OR 2.21; 95% CI, 1.06–4.63; *p* = 0.030, respectively) [71]. 

## 5. Testicular Cancer Genetics

A study of 205 patients with TGCT found that 9.8% of patients had *CHEK2* mutations and, compared to historical controls, patients with TGCT were significantly more likely to carry germline *CHEK2* alteration (OR > 1.4; *p* = 0.03) [72]. Additionally, carriers of *CHEK2* mutations developed TGCTs almost six years earlier than those with TGCTs and wild-type *CHEK2* (5.95 years; 95% CI, 1.48–10.42; *p* = 0.009 [72]. An analysis of 137 TGCTs identified three somatic mutations that were significantly altered in these patients: *KIT* (18%), *KRAS* (14%), and *NRAS* (4%), all of which are potentially actionable with targeted therapeutics. *KRAS* was further validated in a 47-patient study as the most frequently altered gene [73,74]. These findings help elucidate the biology of TGCT, define genomic drivers of pathogenesis, and offer potentially actionable therapeutic targets.

The most commonly altered genes in TGCT identified are *KIT*, *TP53*, *KRAS*/*NRAS*, and *BRAF* [75]. Further, one study found that there were more mutations in *KIT* in patients with bilateral TGCT compared with unilateral disease (93% vs 1.3%) [76]. One study found *NRAS* to be mutated in up to 65% of TGCT [77]. Additionally, *BRAF* V600E mutations were associated with chemoresistance, with 26% of cisplatin resistant TGCTs harboring *BRAF* V600E mutations comparing with only 1% in the cisplatin sensitive TGCTs [78].

In order to better identify the genomic landscape of TGCT, a five-patient phase II study analyzed targeted exome sequencing data of platinum-refractory TGCT who were treated with sunitinib. In this study, one patient had a progression-free survival of 17 months and was found to have *RET* amplification, *PTEN* loss, *EGFR* and *KRAS* amplifications [79]. Of these genes, the *RET* amplification was believed to be the driver mutation targeted by sunitinib, resulting in a profound response [79]. c-KIT mutations in TGCT may confer response to imatinib, but the clinical utility of this therapy for TGCT remains controversial [80]. 

## 6. Analysis of The Cancer Genome Atlas (TCGA)—Last Accessed 13 March 2020

**Figure 2:**
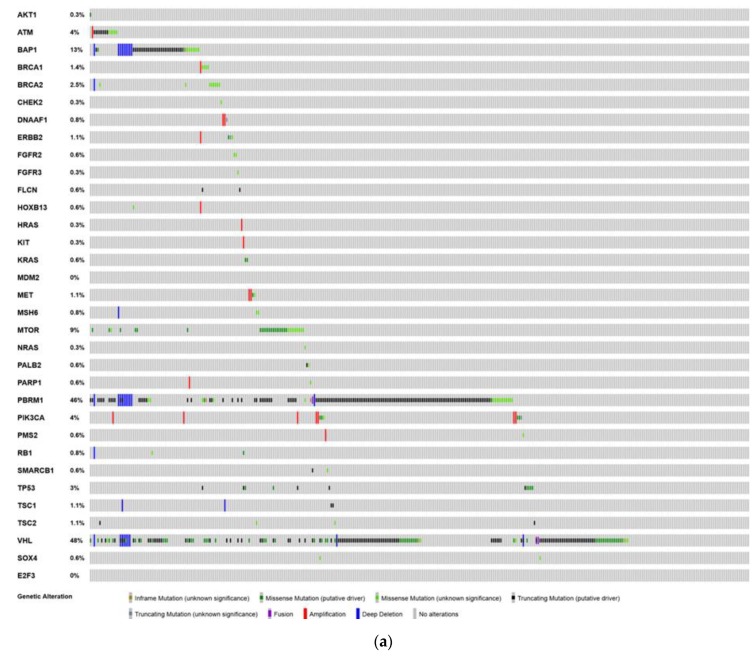

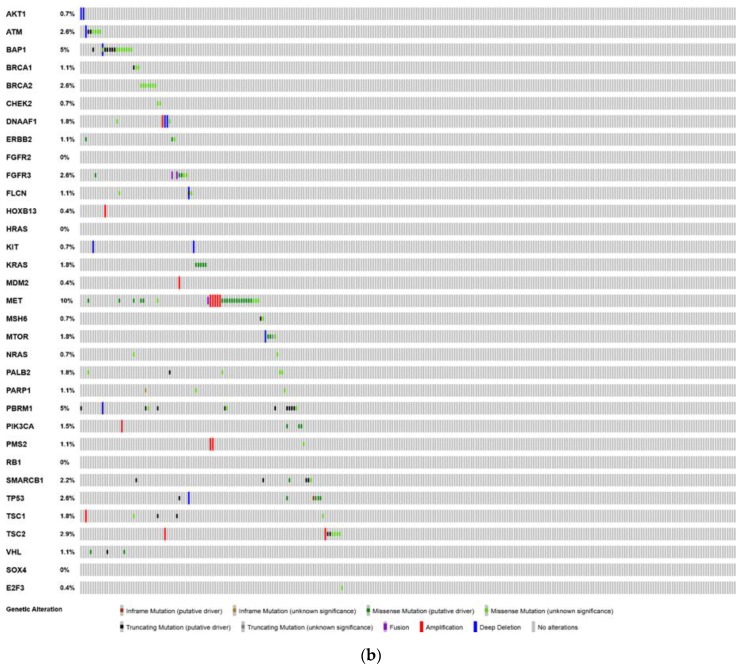

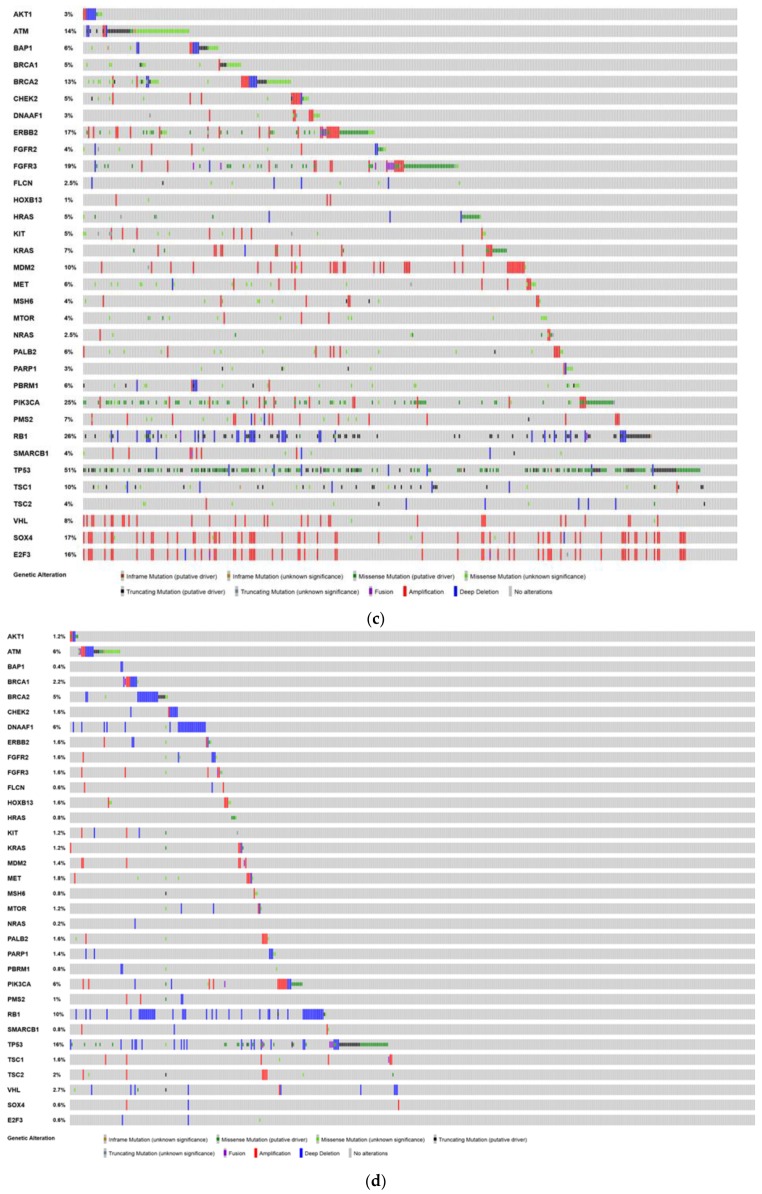

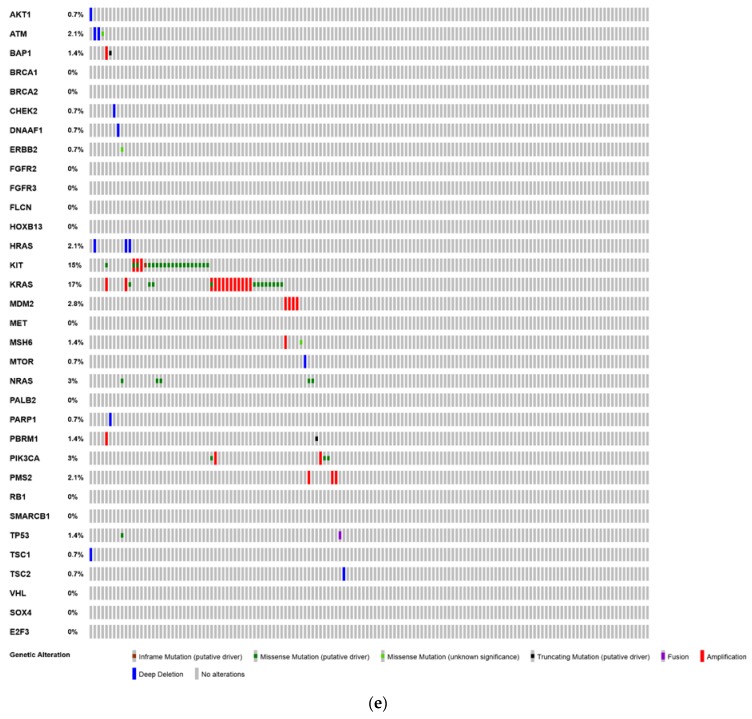
The Cancer Genome Atlas (TCGA) figure was generated to show copy number alterations, and mutations in selected genes. The figure panel was created using the cBioPortal [81] for the bladder, kidney (papillary and clear cell), bladder urothelial, and prostate data set available on the portal. Deep deletion denotes a deep copy number loss, potentially a homozygous deletion. Amplification denotes a high-level, often focal, copy number gain of multiple copies. Deep deletions and amplification are considered more biologically relevant than shallow deletions (which are often heterozygous) and low-level copy number gains. (**a**). clear cell renal cell carcinoma TCGA mutational landscape. (**b**). papillary renal cell carcinoma TCGA mutational landscape. (**c**). Bladder Urothelial Carcinoma TCGA mutational landscape. (**d**). Prostate adenocarcinoma TCGA mutational landscape. (**e**). Testicular cancer TCGA mutational landscape.

We analyzed next-generation sequencing data from the Cancer Genome Atlas (TCGA) in an effort to elucidate the genomic landscape of these tumors (Figure 2). The figures were generated to show copy number alterations and mutations in selected genes. The figure panel was created using the cBioPortal [81] for the kidney (papillary [n = 274], clear cell [n = 354]), bladder urothelial (n = 406), prostate adenocarcinoma (n = 489), and testicular cancer (n = 144) data sets available on the portal. Patients with prostate adenocarcinoma had a higher frequency of mutations in genes related to DNA repair: *BRCA2* (5%), *CHEK2* (1.6%), *ATM* (6%), as well as mutations in cell cycle regulating genes: *RB1* (10%) and *TP53* (16%). The papillary RCC dataset showed high mutation rates in the *MET* gene (10%), which is targeted with MET inhibitors like cabozantinib [82] as well as mutations in *PBRM1* (5%) which can affect responses to immunotherapy [83]. The urothelial carcinoma dataset showed mutations in targetable genes such as *FGFR3* (19%), *ATM* (14%), *BRCA2* (13%), *PIK3CA* (25%), and *ERBB2* (17%) which may provide insight into future therapeutic strategies. The most commonly found mutation in the TCGT sample was *KRAS* (17%) followed by *KIT* (15%), which are both targetable and may affect platinum chemosensitivity and disease progression.

## 7. Genitourinary Genetic Counseling for the Practicing Physician

### 7.1. Genetic Counseling Overview

While it has been well established that there are a number of germline mutations associated with an increased risk of urologic malignancy, genetic counseling is an often-underutilized component of the work-up for cancer patients. Significant factors contributing to the underutilization of genetic testing are likely the lack of clear guidelines regarding how the results should be used to alter management and the shortage of genetic counselors. However, improvements are continuously being made [84,85,86]. Still, there remains a significant amount of work needed to better determine which patients should undergo genetic testing, the timeframe during which testing should be conducted, what techniques should be used, and how the information can be utilized to better serve patients and their families. Additionally, the decreasing cost and increasing number of available genetic tests has likely contributed to the demand for genetic counseling, which further exacerbates the lack of genetic counselors [84].

Genetic counseling is a field that helps patients navigate the complexities and implications of genetic testing, as genetic testing results can have significant medical, psychological, and familial consequences that patients are unsure how to address. It requires training in medical genetics through an Accreditation Council for Genetic Counseling (ACGC) accredited master’s program and passing the American Board of Genetic Counseling (ABGC) certification exam [87]. As of 2017, there were just over 4200 certified genetic counselors in the US and that number is expected to increase over the coming years. However, there appears to be a lack of genetic counselors when compared to the demand, making it difficult for many patients to receive proper genetic counseling [88].

One manner in which this shortage may be addressed is by trained surgeons or medical oncologists taking a more proactive role in counseling patients about genetic testing. A possible arrangement could have oncologists performing the initial pre-test genetic counseling (i.e., explaining risks/benefits of testing, why patient should have testing), while patients with pathogenic mutations or variations of unknown significance (VUS) receive more thorough genetic counseling from a genetic counselor. This arrangement was recently assessed during the ENGAGE (Evaluating Streamlined Onco-genetic BRCA Testing and Counseling Model Among Patients with Ovarian Cancer) study where patients with ovarian cancer underwent BRCA mutation testing. The study demonstrated high rates of patient and oncologist satisfaction with the aforementioned model [89]. It is important to note this arrangement requires oncologists to have undergone adequate training in pre-test counseling, and for there to be a close partnership between oncologists and genetic counselors to ensure patients are receiving high-quality and timely care. One particular patient population that has been shown to receive suboptimal care at times is patients with genetic results demonstrating VUS. In a study where patients with breast cancer underwent BRCA testing, it was found that nearly half of the surgeons involved did not understand the difference between VUS and pathogenic mutations [90]. This is significant because a number of the patients with VUS underwent bilateral mastectomy, even though the procedure has only shown a survival benefit for those with a known pathogenic variant of the BRCA gene [91,92,93]. While these patients did not have urologic malignancy, this serves as an example of harm that can be done to patients if physicians do not understand the significance of genetic testing results.

### 7.2. Genetic Testing and Prostate Cancer

Of the urologic malignancies, genetic testing is likely to impact the management of prostate cancer more than others, namely due to the potential use of targeted therapies for patients with known germline mutations [86]. Currently, the National Comprehensive Cancer Network (NCCN) guidelines state patients with metastatic prostate cancer, patients with a Gleason score ≥ 7 and a family history suspicious for possible high-risk germline mutations should undergo genetic counseling and consider genetic testing [94]. It is also worth noting that the NCCN guidelines state genetic testing is likely to be low-yield in patients with no family history suggestive of high-risk germline mutations, or prostate cancer with no suspicious clinical features (i.e., high or very-high risk prostate cancer, intraductal histology) [94]. Moreover, it is important the appropriate patents are selected for genetic testing as the results may cause unnecessary stress for patients, especially if testing shows VUS.

As to how the discovery of a pathogenic mutation can impact prostate cancer screening, the NCCN Prostate Cancer Early Detection Guidelines state men with known BRCA1/2 mutations should consider PSA screening at age 40, as opposed to age 45 for average-risk men, following a discussion of risk and benefits. The NCCN states it is also reasonable to conduct repeat screening on an annual basis, regardless of the initial PSA value. There are no specific recommendations in regard to other germline mutations. Additionally, the NCCN states a PSA ≥ 3 ng/mL should be used as a cutoff for prostate biopsy (the same as their recommendation for men without pathogenic germline mutations) as there is not enough evidence to support a change in PSA cutoffs at this time. Currently, the IMPACT study is being conducted to aid the development of early detection guidelines for prostate cancer in men with BRCA1/2 germline mutations. Following the first round of screening, it has shown no difference between BRCA1/2 mutation carriers and controls in the rate of detection of prostate cancer or the positive predictive value of prostate biopsy in men with a PSA ≥ 3 ng/mL. However, no conclusions can be made until more follow-up data are collected [95].

### 7.3. Genetic Testing and Renal Cancer

As aforementioned, there is a multitude of hereditary syndromes related to increased risk of kidney cancer. Many of these syndromes have extrarenal manifestations that can cause significant morbidity and mortality if not properly managed; hence, identifying patients with these conditions can have major implications for management of the patient and their family members. As far as who should be referred for genetic counseling, the NCCN and American Urological Association (AUA) recommend patients who are 46 years of age or younger with renal malignancy undergo genetic counseling [96,97]. The AUA also states genetic counseling should be considered for patients with multifocal or bilateral renal masses, or a personal or family history suggestive of hereditary renal neoplastic syndromes. Moreover, it is worth nothing these statements are considered “expert opinion” [97].

In patients who have family members with a known hereditary kidney cancer germline mutation, the age to start screening depends on the particular mutation. If it is a syndrome in which kidney cancer presents during adulthood, a discussion about screening can take place when the patient turns 18. However, if the syndrome is associated with childhood kidney cancer, screening during childhood should be considered [98]. At this time, the optimal screening modality for kidney cancer has not been determined, but urine dipstick, biomarkers, renal ultrasound, and abdominal CT scans have been used with various levels of success [99].

### 7.4. Genetic Testing and Upper Tract Urothelial Carcinoma

In addition to prostate and renal cancer, upper tract urothelial carcinoma (UTUC) can be linked to germline alterations. Specifically, Lynch syndrome is highly associated with UTUC, and is the most common inherited cancer syndrome. Lynch syndrome occurs due to defects in the DNA mismatch repair (MMR) system resulting in microsatellite instability (MSI), and ultimately leading to various types of cancer [100]. Per the European Association of Urology (EAU), the possibility UTUC is a manifestation of Lynch syndrome should be investigated with genetic testing in patients less than 60 years of age with hereditary nonpolyposis colorectal cancer (HNPCC) spectrum cancer, patients less than 60 years old with a 1st degree relative younger than 50 years old with a HNPCC-spectrum cancer, and patients less than 60 years old with two first degree relatives with HNPCC-spectrum cancers (i.e., colon, small bowel, stomach, pancreas, endometrium ovary, bladder) [101]. Moreover, the revised Bethesda guidelines can be used to select patients without UTUC who should be tested for Lynch syndrome [102].

The diagnosis of Lynch syndrome requires tissue testing and germline genetic testing [100]. If tumor tissue is available, immunohistochemistry and MSI-PCR should be performed to evaluate for the lack of MMR proteins and amount of MSI, respectively. Should these tests demonstrate a patient is at high risk for Lynch syndrome, through either a lack of proper MMR proteins (i.e., MLH1, MSH2, MSH6, or PMS2) or MSI-high, they should undergo germline genetic testing to confirm the diagnosis of Lynch syndrome [103]. Lastly, there is no clear consensus regarding how patients with Lynch Syndrome should be screened for urinary tract carcinoma. The EAU guidelines states patients with Lynch syndrome do not need to be screened for urinary tract cancer, while US guidelines say they can be screened for microscopic hematuria starting at age 30–35 [104,105].

## 8. Conclusions

Understanding the diverse genomic aberrations that lead to GU malignancies can guide the development of targeted therapeutic strategies. The key driver mutations in familial syndromic cancers have informed studies of key biological pathways. In RCC, well-described hereditary syndromes have served as real-time clinical models for the pathogenesis of sporadic cases with similar somatic gene alterations. Common bladder cancer genetic mutations can be targeted by rationally designed therapies. In prostate cancer, the large patient population has provided valuable data of genomic correlates for increased disease risk that can inform potential screening strategies. The discovery of new molecular markers in testicular cancer may also guide novel treatment strategies for the subset of patients with relapsed/refractory disease to cytotoxic chemotherapy. A better understanding of the molecular pathogenesis of urologic malignancies can expand the therapeutic armamentarium against these diseases and improve the outcomes of patients who are resistant to currently approved regimens.
